# A 11 mW 2.4 GHz 0.18 µm CMOS Transceivers for Wireless Sensor Networks

**DOI:** 10.3390/s17020223

**Published:** 2017-01-24

**Authors:** Bing Hou, Hua Chen, Zhiyu Wang, Jiongjiong Mo, Junli Chen, Faxin Yu, Wenbo Wang

**Affiliations:** 1 School of Information and Communication Engineering, Beijing University of Posts and Telecommunications, Beijing 100876, China; hanchen_hb@163.com (B.H.); wbwang@bupt.edu.cn (W.W.); 2 School of Aeronautics and Astronautics, Zhejiang University, Hangzhou 310027, China; chenhua@zju.edu.cn (H.C.); jiongjiongmo@zju.edu.cn (J.M.); fxyu@zju.edu.cn (F.Y.); 3 School of Electronic Information and Electrical Engineering, Shanghai Jiao Tong University, Shanghai 200240, China; chen09068@163.com

**Keywords:** wireless sensor networks, RF transceiver, receiver, transmitter, frequency synthesizer, CMOS RF IC

## Abstract

In this paper, a low power transceiver for wireless sensor networks (WSN) is proposed. The system is designed with fully functional blocks including a receiver, a fractional-N frequency synthesizer, and a class-E transmitter, and it is optimized with a good balance among output power, sensitivity, power consumption, and silicon area. A transmitter and receiver (TX-RX) shared input-output matching network is used so that only one off-chip inductor is needed in the system. The power and area efficiency-oriented, fully-integrated frequency synthesizer is able to provide programmable output frequencies in the 2.4 GHz range while occupying a small silicon area. Implemented in a standard 0.18 μm RF Complementary Metal Oxide Semiconductor (CMOS) technology, the whole transceiver occupies a chip area of 0.5 mm^2^ (1.2 mm^2^ including bonding pads for a QFN package). Measurement results suggest that the design is able to work at amplitude shift keying (ASK)/on-off-keying (OOK) and FSK modes with up to 500 kbps data rate. With an input sensitivity of −60 dBm and an output power of 3 dBm, the receiver, transmitter and frequency synthesizer consumes 2.3 mW, 4.8 mW, and 3.9 mW from a 1.8 V supply voltage, respectively.

## 1. Introduction

Short-distance communications have recently spurred lots of researches and developments. The RF transceiver is undoubtedly one of the key components in a communication system [1,2,3,4,5]. However, it is not possible to design a transceiver to host all types of communication systems. Depending on the applications, the topologies of transceiver ICs or systems may vary considerably in terms of the system architecture and performances such as data-rate, power consumption, physical dimensions, etc. [1,2,3,4,5,6,7,8,9,10,11]. For example, Complementary Metal Oxide Semiconductor (CMOS) RF transceivers for short-range communications have been reported extensively, but it is still of a great importance to design transceivers for certain applications since no one solution can cover all the desires in different scenarios where the design strategies may vary significantly. To achieve ultra-low power consumption, many transceivers are designed without low noise amplifier (LNA) or voltage controlled oscillator (VCO) [4,10] at the expense of functionality. For an oscillator-less design or injection-locked oscillator-based architecture, the transceivers are unable to provide programmable channel frequencies, which limit the functionality of the system. The functionality of such a system are quite limited even though sub-mW operation is achieved [10]. For example, in the applications of wireless sensor networks (WSN), the design considerations are quite different even though low power consumption is always a key advantage. The amount of chips used in WSN applications will be massive, and there are many different protocols and communication standards involved in such applications that it is, therefore, highly desired that the transceiver is designed with fully functional blocks, such as a LNA, phase-locked loop (PLL), and the cost of the circuits should be as low as possible. For example, the design will be attractive if all of the circuits can be implemented with low cost but reliable technology, such as 0.18 μm CMOS technology, which maintains a good balance among the level of integration, cost, and performance. Of course, the designs should have fewer off-chip components and a small silicon area. Therefore, the number of on-chip inductors or large capacitors (higher than tens of pF) should be kept as low as possible.

By considering all of these factors, this paper presents a low power transceiver working on the 2.4 GHz band which is one of the most popular Industrial Scientific Medical (ISM) communication bands. The system is designed with conventional architecture, but it is optimized to maintain good balance among the considerations in WSN applications. This paper is organized as follows: Section 2 presents the system planning of proposed transceiver, followed by the implementation of circuits in Section 3; Section 4 demonstrates the experimental results of the proposed circuits and system; and Section 5 is the conclusion.

## 2. System-Level Design of a Short-Range Transceiver

The architecture of a transceiver greatly depends on the applications of the system. For short-range applications, the data rate and the distance of the transferring signal are quite limited, hence, the key considerations are low power consumption and small silicon area (low cost). The link budget, as well as the sensitivity of the receiver, are given by [4]:

P_RX,in_ = P_TX,out_ − 20log(4dπ/λ) (1)

Sens_RX_ = NoiseFloor + 10logBW + NF + SNR_out_ (2)

where P_TX,out_ is the output power of the transmitter, d is the distance of communication, and λ is the wave length of the carrier. Since the distance of communication is at metre level, a lower output power of PA or received power of RX is applicable. As illustrated in [4], at the distance within one metre, even a −40 dBm received power, is possible with a 40 GHz carrier. The requirement of received power can be even relaxed for a 2.4 GHz carrier, which has with much less attenuation of propagation. Moreover, as shown in Equation (2), for a fixed noise floor, to accommodate a lower data rate transfer, it is possible to use a narrow signal bandwidth and a simpler modulation scheme, hence it is possible to make compromises in system specifications such as sensitivity or noise figure.

**Figure 1:**
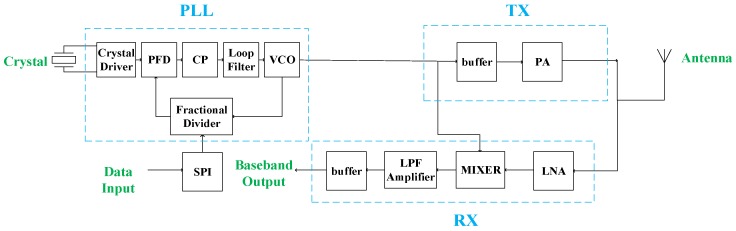
The architecture of the proposed work.

Figure 1 shows the architecture of the proposed system. It includes three key sub-systems, namely, the receiver (RX), phase-locked loop (PLL), and transmitter (TX). The receiver includes a low noise amplifier, mixer, low pass filter with an amplifier stage (IF amplifier), and the buffer for test purposes. The local oscillator (LO) signal is provided by a PLL, which includes a crystal driver as the reference clock, a phase-frequency detector (PFD), charge pump (CP), loop filter, voltage controlled oscillator (VCO), and a fractional-N divider. The output signal of the PLL is also used as the input signal of power amplifier (PA) after a buffer stage, which generates the frequency shift keying (FSK) or amplitude shift keying (ASK)/on-off-keying (OOK) output signals of the TX. When the TX is working in ASK/OOK mode, the input of the TX (output of the PLL) is fixed to a certain frequency, while the biasing of the PA is modulated by the input DATA signal as an on-off (high gain or low gain) state. Thus, the transceiver performs a dual-mode operation. The target performances of this system are determined as follows, based on the above-mentioned strategy.

The system is designed using a 0.18 µm CMOS technology, which is a popular solution for industrial applications. For simplicity, the transceiver is designed to support FSK and ASK systems since the data rate requirement is quite low (usually considerably lower than 1 Mbps) in a typical WSN system. If more complex modulation schemes are needed, digital baseband systems can be added in this proposed work. The specifications of the subsystem can be determined based on the operating frequency and technology in use since many individual building blocks have been reported in the literature [12]. In this scenario, the maximum noise figure of the receiver can be as high as 20 dB [12]. This makes it possible to achieve a low power consumption in the design of the RX (mW level). At a distance of around 10 m, the output power of 0 dBm or less is a reasonable choice, even higher output power is possible with CMOS technology [11]. For a power amplifier with a power efficiency of 20%, the DC power consumption of the TX will be less than 5 mW [12]. To improve the performance, an accurate LO is needed, even though the requirement of phase noise performance is not a difficult one to meet. For example, −96 dBc/Hz at 1 MHz offset will be enough in such a system [12].

## 3. Circuit Implementation

### 3.1. Low Noise Amplifier and Power Amplifier

**Figure 2:**
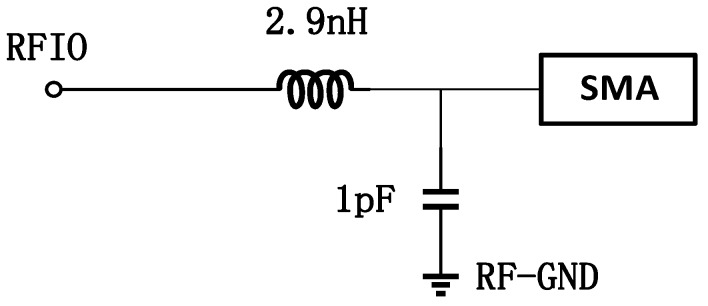
The off-chip matching network of the RFIO.

As illustrated in [12], since the TX and RX share an antenna, the PA and LNA can be co-designed to share the matching network. The basic consideration is that when the LNA is in the off mode, the system is working as a transmitter. However, the LNA simultaneously contributes parasitic capacitance. Similarly, in the RX mode when the PA is powered off, the parasitic capacitance of the PA should be included in the simulation of LNA. The off-chip connection of the LNA/PA to the antenna is shown in Figure 2.

**Figure 3:**
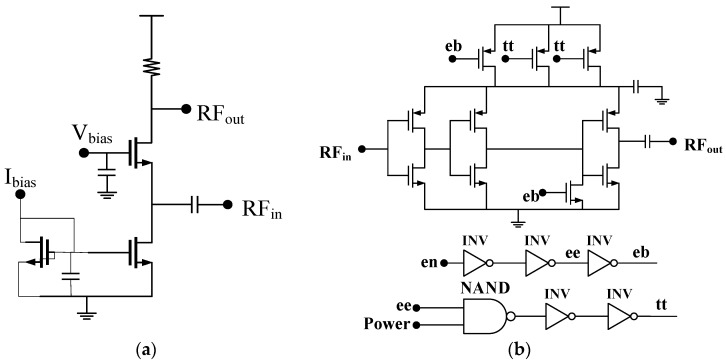
Topology of the LNA and PA (**a**) low noise amplifier; and (**b**) PA with control circuit.

Figure 3 shows the topology of the low noise amplifier and the power amplifier. It is an inductor reuse topology proposed in [12]. As mentioned above, the key considerations in this transceiver are low cost (small silicon area) and flexibility in functionality subject to acceptable performances, such as noise figure, gain, and linearity. Hence, it is necessary to maintain less off-chip component and small silicon area. Additionally, it would be more attractive if the transceiver is able to be re-configured for different applications, since the WSN applications cover a wide range of operating frequencies. Thus, the strategy in this design is that the RX and TX are inductor-less and only one off-chip inductor is shared by the RX and TX. The silicon area of the transceiver will, therefore, be ultra-small. Moreover, since the matching network is determined by the inductor and capacitor, it will be very convenient to make the RX and TX work properly at the desired frequency range.

**Figure 4:**
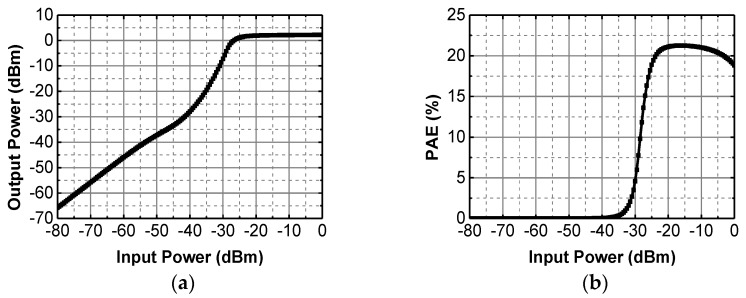
The simulation results of the PA (**a**) output power; and (**b**) PAE.

The PA in this design is a conventional class-E topology as shown in Figure 3. It consists of three stages of inverters, the output stage has an output matching network with an on-chip capacitance and an off-chip inductor with capacitances, which forms a PI-matching topology. This topology, which is widely used in short-range communication exhibits a high efficiency and small silicon area. Additionally, the external matching network make it possible to re-configure the system at different working frequencies. Figure 4 shows the simulation results of the PA, the maximum output power reaches 2.1 dBm, while the power added efficiency (PAE) is 21.2%.

Similarly, the LNA is designed to maintain a good balance among the required performances. To achieve a wide operating band, a common gate topology is implemented since a higher noise figure is acceptable in this system. The parallel resonant circuit shared with the power amplifier forms a band-pass frequency response which improves the robustness against interferences. Another important trade-off is the load of the amplifier. A resistive load, instead of a commonly-used inductive load, is implemented in this design in considering a small silicon area. The drawback of this design is the lower high-frequency gain, sizing of the transistors and resistors is, thus, carried out to ensure acceptable performances in noise figure, gain, and linearity at desired operating frequencies.

**Figure 5:**
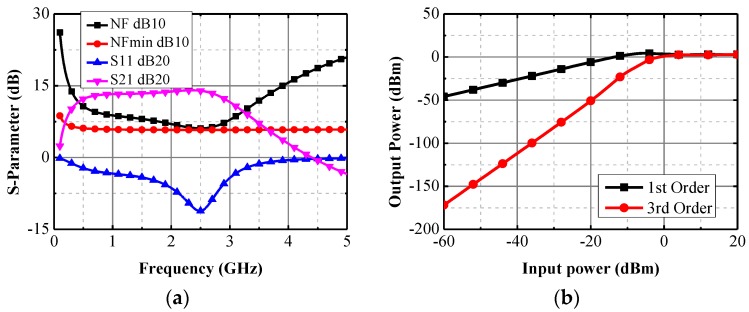
Simulation results of the LNA (**a**) S parameters and noise figure; and (**b**) IP3.

Figure 5 shows the simulation results of the low noise amplifier. It has a wide-band of gain performance, which is over 12 dB from several hundred megahertz to 3 GHz. The noise figure of the LNA is about 6–7 dB within the band of interest. The performances are quite acceptable for a complete receiver with a noise figure budget up to 20 dB. The Third Order Intercept Point (IP3) of the amplifier is higher than 0 dBm, which suggests that the RX exhibits good linearity.

### 3.2. Mixer and Analogue Baseband Circuits

**Figure 6:**
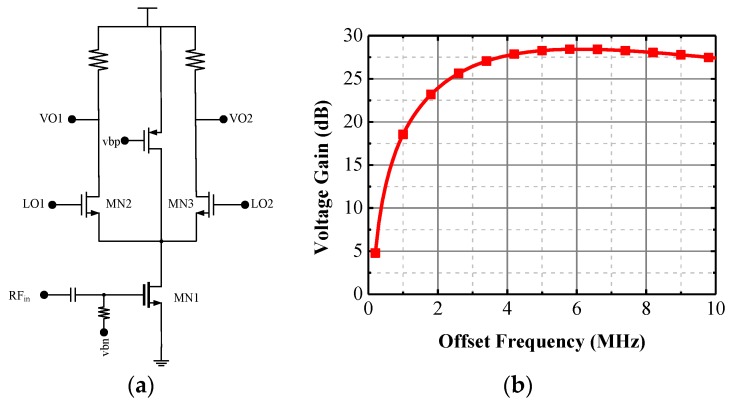
The mixer (**a**) topology and (**b**) simulated gain.

Since the low noise amplifier is single-ended, a single balanced active mixer is used to save power consumption. Shown in Figure 6, the topology in this design is quite conventional and the major task here is the proper sizing of these devices to achieve low power consumption subject to acceptable gain and linearity (the noise figure of the active mixer is not an issue compared with a passive one). The gain of the mixer is linearly proportional to the trans-conductance of the transistors MN2 and MN3 and the reload resistance of the mixer. To save power consumption, it is desirable to use a larger resistance, such as 6 kΩ in this work. Using the biasing current of 0.6 mA, it is possible to achieve over 300 mV output swing and over 15 dB gain at 2.4 GHz to 2.5 MHz (IF) gain. For the linearity, it is mainly decided by the biasing condition of the MOS transistor.

With regard to the low data-rate in this work, the IF frequency is set to 2.5 MHz (reconfigurable). The signal is amplified with an IF amplifier with programmable gain control. The design is a conventional MOS switch-based amplifier [13]. By controlling the biasing condition and switching of different loads. The amplifier has a tuning range of 30 dB in gain.

### 3.3. Frequency Synthesizer

The frequency synthesizer has been a major bottleneck of fully-integrated transceivers [14,15]. Due to this large silicon area and power consumption, it is usually an off-chip sub-system in many ultra-low-power solutions. However, in this work, to ensure the robustness and maximized flexibility, it is designed as a fully-integrated sub-system which provides frequency outputs fractional to the reference clock. It includes a voltage-controlled oscillator, a fractional-N frequency divider, a phase-frequency detector, a charge pump and a loop filter as shown in Figure 1.

**Figure 7:**
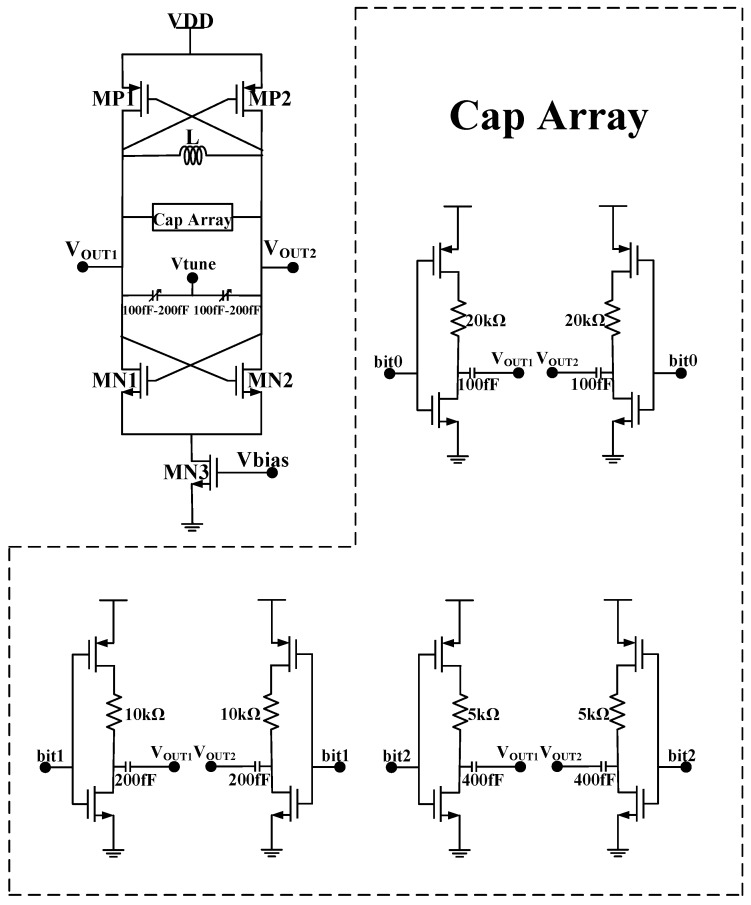
The topology of the VCO.

**Figure 8:**
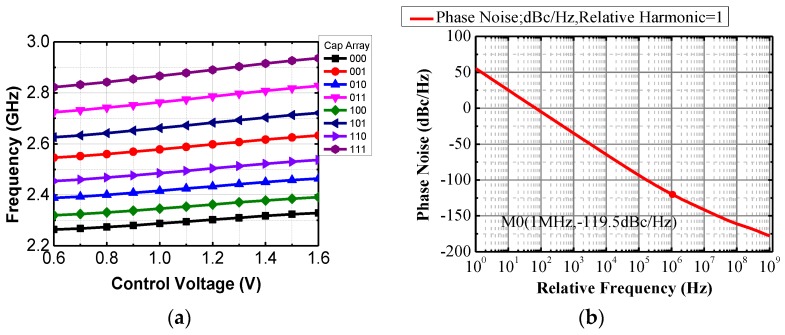
Simulated F-V curve and phase noise of the VCO; (**a**) F-V curve; and (**b**) phase noise at 2.45 GHz.

The voltage-controlled oscillator is the key building block in terms of working range, phase noise, and output power of the PLL. The key considerations are acceptable phase noise with low power consumption and small silicon area. To improve the power efficiency, the PMOS-NMOS cross-coupled negative-G_m_ LC oscillator is used as shown in Figure 7. The optimization is mainly focused on the high-performance LC tank. The oscillator has a start-up condition which is determined by the quality of the tank and the negative G_m_ to compensate the tank loss, which suggests that low power is only possible for a high quality factor (Q) tank. At 2.4 GHz range, the low quality factor of inductor is the major concern. A high Q can be obtained using an off-chip inductor, but it is susceptible to additional parasitic effects in the package and on the PCB. In this work, properly sizing of this on-chip fully-symmetrical inductor is used to ensure a high-quality factor (around 10) at the desired frequency range. The overall quality factor of the LC tank is about 6–7. After optimization, the biasing current of 0.7 mA is able to ensure a robust operation of the oscillator (three times higher than the start-up condition in G_m_). In additional to the varactors for continuous frequency tuning, a three-bit digitally-controlled capacitance bank is implemented so that the proposed VCO can work properly from 2.2 GHz to 2.6 GHz. The gain of the VCO (K_VCO_) is designed to be less than 100 MHz/V to maintain a good phase noise performance, while covering the 2.4 GHz operating band with certain redundancy. The output power of this VCO (with an output buffer) is around 3 dBm. The simulated F-V curve of the VCO is shown in Figure 8. As shown in Figure 8, the simulated phase noise of the VCO is −113 dBc/Hz at 1 MHz offset.

**Figure 9:**
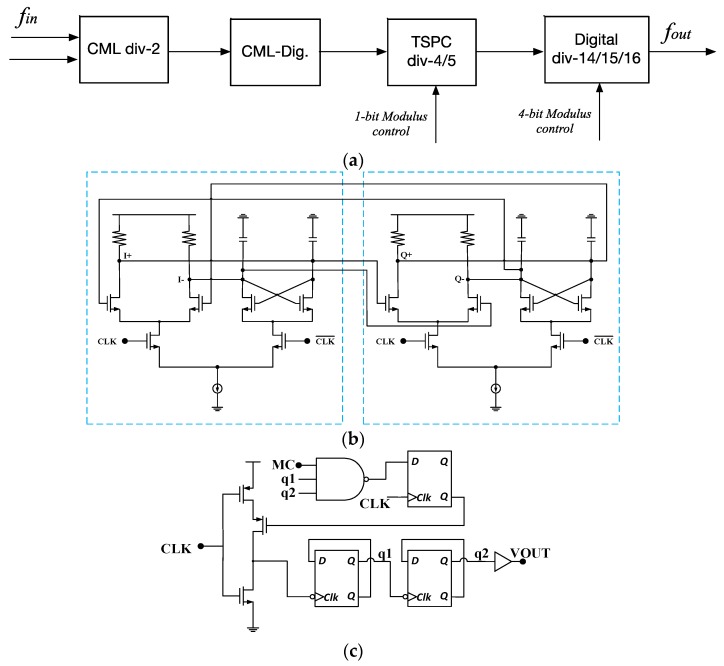
(**a**) The frequency divider; (**b**) CML divide-by-two circuit; and (**c**) divide-by-4/5 circuit.

Another key building block is the frequency divider. As shown in Figure 9, it includes a divide-by-two prescaler, a divide-by-4/5 prescaler, and the P counter with division ratios of 14, 15, and 16. The total division ratio is 2 × (52 + M), where is M is fractional number, which is controlled by a 10-bit sigma-delta modulator (configured as Mash 1-1-1); considering the short-range communication with low power and small silicon area, even the fractional spur can be quite high due the insufficient random dithering. Another key consideration is the power consumption [14]. In this work, the blocks in the frequency divider are designed to be power efficient at the certain working frequencies. The prescaler is the most challenging building block in the frequency divider since it works at the highest operating frequency. The MOS current mode logic (MCML) is the most popular solution at the operating frequency above 1 GHz. However, it is quite power consuming compared with dynamic digital logic such as true-single-phase-clock (TSPC). Therefore, it is preferred that the MCML circuit is only implemented at the highest operating frequency, while the lower frequency division is performed with single-ended logic. In this work, to maintain a good balance between operating frequency and power consumption, the input signal is firstly divided by two using a MCML divider followed by a differential-to-single-ended buffer (CML-Dig in Figure 9). The MCML divide-by-two unit is shown in Figure 8b, which consists of two D-latches. The voltage swings of the input and output of the divide-by-two unit are about 600 mV (peak-to-peak). After a MCML-to-digital buffer, which is a differential to single-ended unit, the output becomes a rail-to-rail signal. Since the output frequency is now about 1.2 GHz, it is possible to use more power efficient logic, namely the TSPC logic in the following divide-by-4/5 prescaler, which is shown in Figure 9c. The D flip-flop used in this design is the conventional nine-transistor TSPC topology [13]. Finally, the output of the divide-by-4/5 prescaler is less than 400 MHz so that the following frequency division can be performed by the divide-by-14/15/16 counter implemented using standard static CMOS logic, which is stable and easy to implement. The total division ratios are determined by four bits of modulus control signals, which are connected to a sigma-delta modulator. In this system, the modulator is of three stages (in Mash 1-1-1) each of which is a 10-bit configuration. The total division ratio is set to be (2 × (52 + M)), where M is a programmable fraction number. Using this fractional-N topology, it is able to achieve division ratios which is a fractional, instead of an integer, number. It is now possible to use a higher reference clock for the PLL system, which results in fast settling time and maximized functionality.

**Figure 10:**
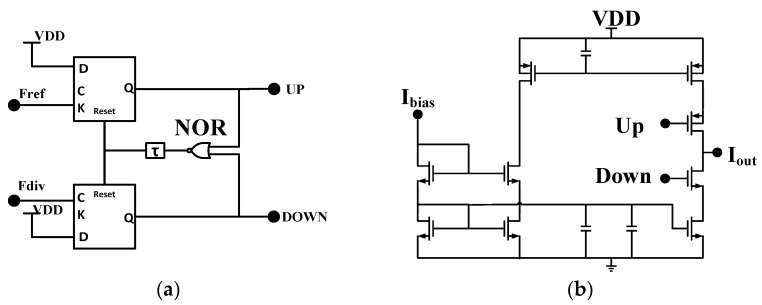

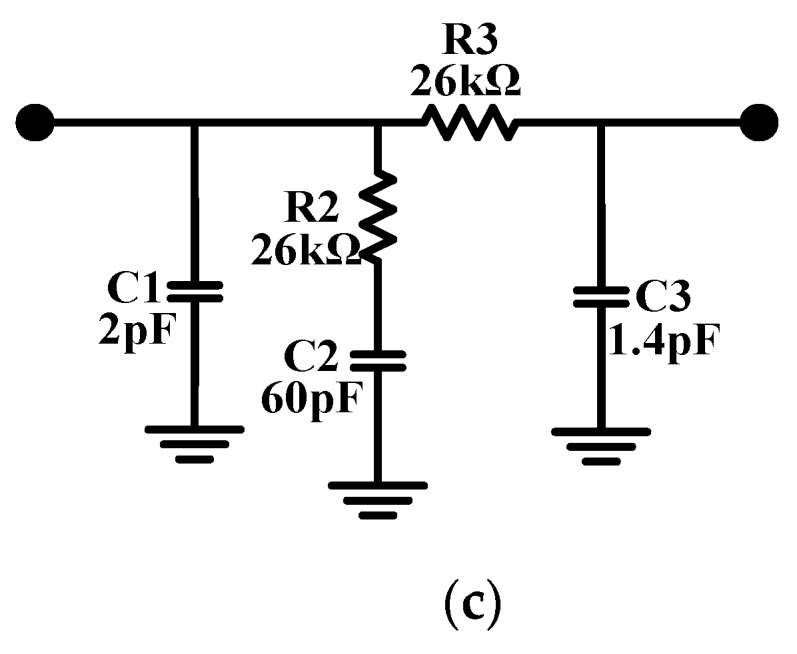
The topology of the (**a**) PFD, (**b**) CP, and (**c**) loop filter.

The other building blocks in the PLL, as shown in Figure 10, are quite conventional. The PFD is a dead zone-free digital detector and the charge-pump (CP) is a conventional one, as shown in Figure 10. The charge current is about 100 μA. A third-order RC-based loop-filter is used for better suppression of the reference spurs. In this work, key consideration is that the capacitor should be small enough to have a fully-integration solution. The parameters of the components in the loop filter are determined by the closed-loop transfer function of the PLL [15]. The whole PLL system is now a fourth-order system [13]. Since the gain of the VCO frequency tuning, operating frequency, division ratio, and charge current have been determined, the PLL has the freedom in optimization of loop filters. As the key target is the silicon area which is occupied by the loop capacitor. In this design, the maximum capacitance after optimization is 60 pF, which occupies 240 μm × 200 μm. The loop bandwidth is 500 kHz to suppress the Delta Sigma Modulator (DSM) quantization noise.

**Figure 11:**
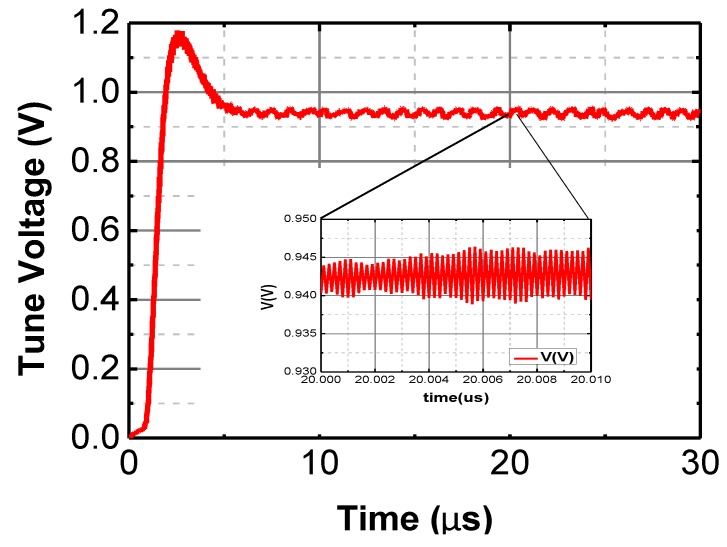
The schematic level simulation result of the PLL.

Simulation of the PLL is carried out using Cadence Spectre RF (San Jose, CA, USA) at the transistor-level in a 0.18 μm CMOS technology (post-layout). Simulation suggests that the proposed PLL is able to work at 2.4 GHz with less than 20 μs settling time, as shown in Figure 11.

### 3.4. System Level Integration

In addition to the key building blocks, there are also some other circuits in the proposed system. The most important part is serial peripheral interface (SPI) circuit, since many circuits in the system are configurable. For example, the transceiver can be configured as an RX or TX, and the gain of the RX is programmable. In addition, by changing the division ratios, which are equal to 2 × (52 + M), the output frequency of the PLL is configurable to support different channels. Accordingly, the working bands of the voltage controlled oscillator should be adjusted to support the desired operating frequencies. The SPI occupies a silicon area of 240 μm × 80 μm (0.0192 mm^2^). The SPI has 32 bits, while 24 of them are used to control the transceiver. D0–D2 are used to control the cap array. D3–D4 control the frequency division of the integer divider. D5–D14 control the frequency division of the fractional divider. D15 is the enable bit of PLL. D16–D19 are used to switch the gain of the IF amplifier. D20 enables the receiver. D21 enables the power amplifier. D22 enables FSK modulate of the transmitter. D23 enables FM modulate of the transmitter. By designing SPI circuit, all of the control bits can be set by several on-chip registers.

Finally, as a complete chip, it includes some functional building blocks, such as the biasing circuits, crystal buffer, and decoupling capacitors. The crystal buffer is designed to support an off-chip crystal of 20 MHz (to meet 2.4 GHz PLL output frequency range). De-coupling capacitors between the supply voltage and ground are added (as many as possible) to improve the noise performance. Implemented using a 0.18 μm CMOS technology, the layout of the transceiver is about 0.7 mm × 0.7 mm and the full chip is about 1.1 mm × 1.1 mm with the added capacitors and ESD pads.

## 4. Measurement Results

**Figure 12:**
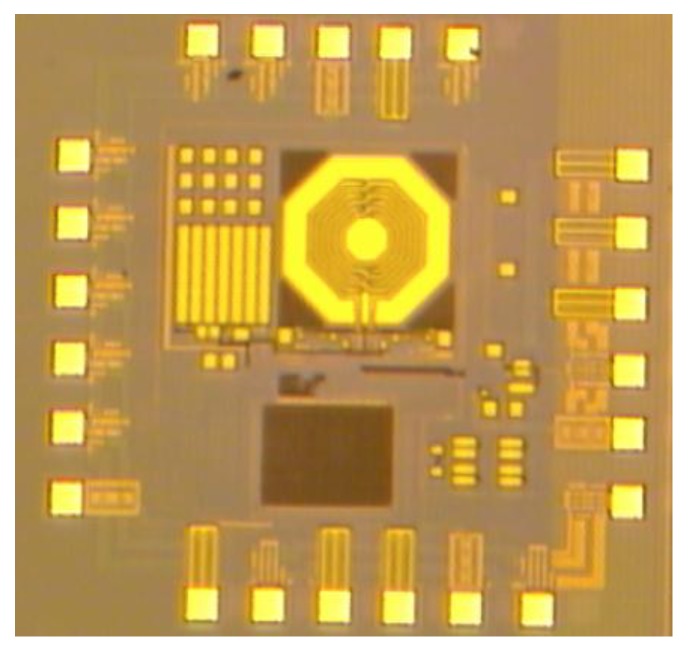
The die photo of the proposed transceiver.

The proposed transceiver is fabricated using a 0.18 μm CMOS technology. Figure 12 shows the die photo of the transceiver, which is packaged in a QFN form for measurements.

The first step of measurement is to set up the initial status of SPI with determines the working conditions of the circuits; for example, the phase-locked loop of this chip. The frequency of the locked PLL is determined by the integer tuning bits (Ch1-0, D11 and D10 of the SPI register) and fractional tuning bits (F9-0, D21-D12 of the SPI register) of the PLL. The output frequency of the PLL, f_out_ is given by:

f_out_ = (59 + Ch1-0 + F9-0/1024) × 2 × 20 MHz (3)

For proper operation of the PLL, one needs to tune the coarse frequency bits of the VCO manually, namely D2–D0 of the SPI register (maybe with the help of a spectrum analyser). The VCO free running frequency should be close to the targeted 2.4–2.5 GHz range.

**Figure 13:**
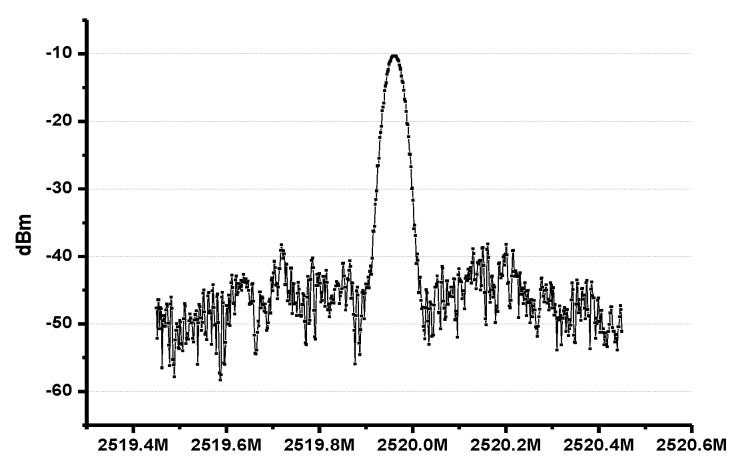
Output spectrum of the PLL.

The matching network for the RFIO port is as follows. The inductance value can be from 2.5 to 3 nH. In this work, the LNA and PA share the matching network with a port namely RFIO as shown in Figure 2. The reference clock of the PLL is 20 MHz, which comes from an off-chip crystal. With the three-bit control of the VCO working bands, the PLL can work from 2.2 GHz to 2.5 GHz properly. Figure 13 shows the output spectrum of the PLL working at 2.5 GHz range.

**Figure 14:**
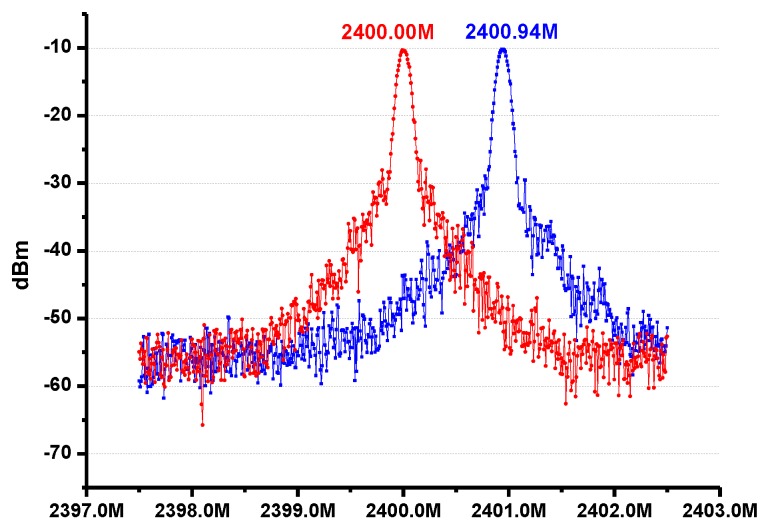
An example of the FSK output spectrum.

As mentioned above, the transceiver supports the working mode of FSK, which is achieved by the changing output frequency in the PLL. The output spectrum of this working mode is shown in Figure 14.

**Figure 15:**
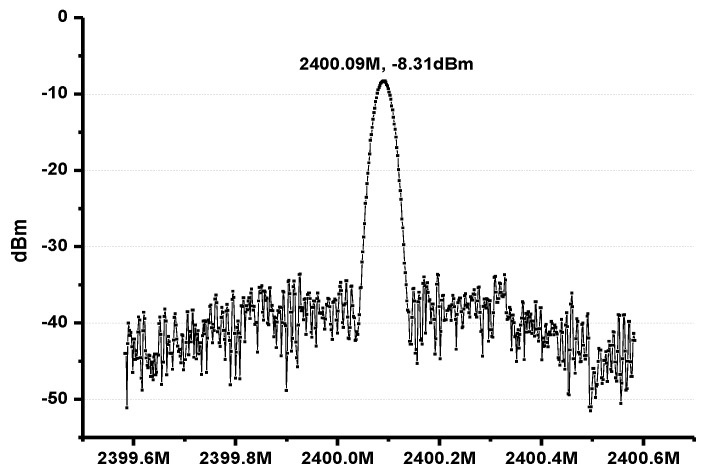
Output spectrum of the PA.

After the verification of the local oscillator, it is possible to connect the output of the PLL to PA, which generates the output of ASK or FSK signal. Figure 15 shows the output spectrum of the PA. The output power is 10 dB lower than the simulation results due to the 0.1× probe in the measurement facilities. If a higher output power level is required, more amplifier stages can be added at the expense of power consumption.

**Figure 16:**
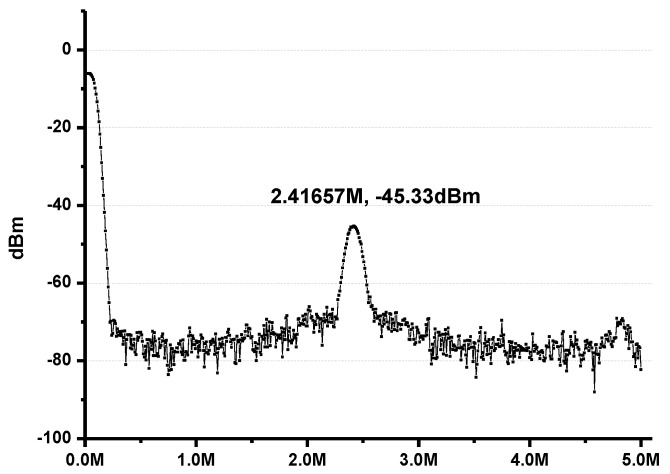
IF signal of the RX.

Finally, this signal is received by the RX, which generates the IF signal at around 2.5 MHz (depending on the configuration). Figure 16 shows the output spectrum of the receiver. The IF amplifier has a tunable gain up to 25 dB to support the dynamic range of the RX.

**Figure 17:**
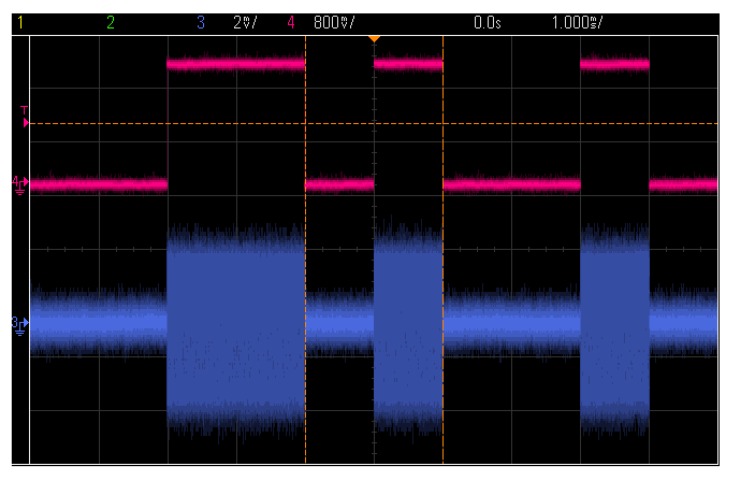
Data in TX (red) and IF in RX (blue).

Based on this analogue performance measurement, it is possible to measure the transceiver using digital signals. Different data patterns, for example, a random generated data of 0001_1111_0101_1011_0111_1100, are used in the transceiver. Figure 17 shows the transmitted data of the TX in OOK modulation (red) and the received IF signal of RX (blue), respectively.

**Figure 18:**
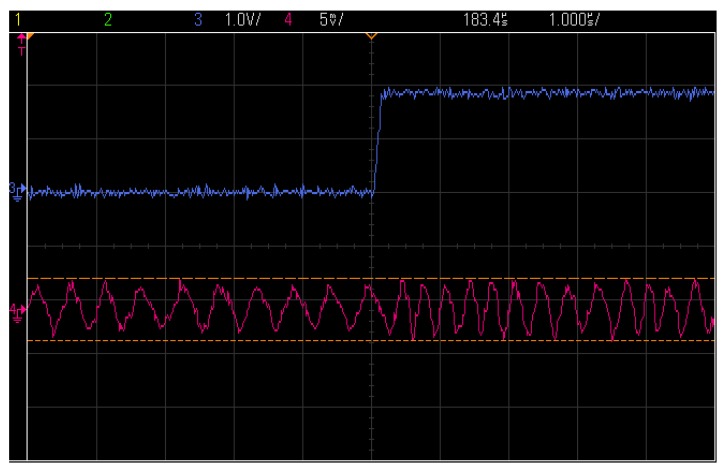
The output signal of an FSK transmission.

Figure 18 shows the data to be modulated and transmitted by TX (blue) and the received IF FSK signal before demodulation (red). By using an FSK demodulator, or comparing to the output IF signal, the data signal can be easily recovered as shown in Figure 18. Measurement results suggest that the proposed transceiver is able to work with 500 Kbps data transferring with mW level power consumption. Table 1 compared the proposed work with some of the recent literatures in similar technology. It is not of the lowest power efficient, but it is based on the standard architecture with a large flexibility in system performances and it can be easily changed to different applications. Thanks to the low cost CMOS technology with small silicon area (only one external inductor is needed), it is of a great interest in the applications of WSN.

## 5 Conclusions

This paper presents a low-cost 2.4 GHz CMOS transceiver. An input-output matching network is shared by the LNA and PA to save silicon and simplify the topology. Power consumption optimization is carried out in the phase-locked loop to maintain a good balance among phase noise and working range. Implemented in standard 0.18 μm CMOS technology, the proposed work is able to work 2.4 GHz with 500 K bps transferring rate, while consumption is 11 mW from a 1.8 V supply voltage.

## Figures and Tables

**Table 1:** Comparison of proposed work with those in the literature.

Transceiver			
Reference	[3]	[8]	This Work
Technology	0.18 μm	0.18 μm	0.18 μm
Supply	1.8 V	1.8 V	1.8 V
Modulation	N/A	QPSK	FSK/OOK
Frequency	2.4	2.4	2.4
Data rate	N/A	2 Mbps	500 Kbps
Power consumption	49.3 mW	17 mW	11 mW
Silicon area	3.92 mm^2^	2.85 mm^2^	1.2 mm^2^ (0.7 without pads)
